# Siglec-7/-9 ligands shield tumor cells from NK cell attack

**DOI:** 10.1186/2051-1426-1-S1-P157

**Published:** 2013-11-07

**Authors:** Camilla Jandus, Kayluz Frias Boligan, Obinna Chijioke, He Liu, Meike Dahlhaus, Thomas Démoulins, Christoph Schneider, Marc Wehrli, Robert E Hunger, Gabriela M Baerlocher, Hans-Uwe Simon, Pedro Romero, Christian Münz, Stephan von Gunten

**Affiliations:** 1 Institute of Pharmacology, University of Bern, Bern, Switzerland; 2 Department of Viral Immunobiology, Institute of Experimental Immunology, University of Zürich, Zürich, Switzerland; 3 Department of Hematology, University Hospital of Bern, Bern, Switzerland; 4 Experimental Hematology, Department of Clinical Research, University of Bern, Bern, Switzerland; 5 Institute of Virology and Immunoprophylaxis, Mittelhäusern, Switzerland; 6 Department of Dermatology, University Hospital of Bern, Bern, Switzerland; 7 Translational Tumor Immunology Group, Ludwig Center for Cancer Research at the University of Lausanne, Lausanne, Switzerland

## 

Altered surface glycosylation on malignant cells may affect tumor immunity by direct interaction with glycan-binding proteins (lectins) on immune cells. Siglec-7 and -9 are MHC class I-independent inhibitory receptors on human NK cells that recognize sialic acid-containing carbohydrates (sialoglycans). We have found that Siglec-7 and -9 ligands are significantly overexpressed on human tumor cell lines of different histological types and in tumor biopsies from melanoma patients. Enzymatic removal of these sialoglycans ligands on tumor cells or interference with blocking monoclonal antibodies (mAbs) to Siglec-7 and -9 conferred in vitro stimulatory NK cell activity (e.g. specific killing, degranulation, cytokine secretion) and considerably greater anti-tumor responses not only to NK cell-sensitive, but also to relatively NK cell non-susceptible tumor cells. In in vivo experiments, in a mouse model with a reconstituted human NK cell compartment, a significant increase in NK cell-mediated cytotoxicity was obtained after removal of sialic acids on the surface of the target tumor cells. The observation that tumor cells use Siglec ligands for shielding against NK-cell attack has direct implications for NK cell-based anti-tumor strategies and for the design of glycan-based cancer therapeutics.

